# Severe Brief Resolved Unexplained Event in a Newborn Infant in Association with Maternal Sertralin Treatment during Pregnancy

**DOI:** 10.3390/medicines5040113

**Published:** 2018-10-22

**Authors:** Mirjam Pocivalnik, Manfred Danda, Berndt Urlesberger, Wolfgang Raith

**Affiliations:** Division of Neonatology, Department of Pediatrics, Medical University of Graz, 8036 Graz, Austria; mirjampocivalnik@hotmail.com (M.P.); manfred.danda@klinikum-graz.at (M.D.); berndt.urlesberger@medunigraz.at (B.U.)

**Keywords:** neonatal abstinence syndrome, selective serotonine reuptake inhibitor, brief resolved unexplained events, apparent life-threatening events

## Abstract

**Background**: Selective serotonin reuptake inhibitors are a very common choice of antidepressive drug-therapy during pregnancy. In up to 30% of cases, they have been found to cause neonatal abstinence syndrome in newborn infants. Although often both time-limiting and self-limiting, severe symptoms of neonatal abstinence syndrome (NAS) can occur. **Methods/Results:** We report a term male infant suffering from a severe brief resolved unexplained event caused by his mother’s sertraline intake during pregnancy. **Conclusions**: Newborn infants exposed to selective serotonine reuptake inhibitors (SSRIs) during pregnancy should be evaluated very carefully concerning NAS and monitored for NAS symptoms for a minimum of 72–96 h, or until symptoms have fully recovered using standardized protocols. There is a risk of severe NAS symptoms which might occur, and this circumstance should be discussed with the parents and taken into account before administering the drug.

## 1. Introduction

Women during their childbearing years are at risk of developing major depression, with a prevalence of up to 13% during pregnancy and up to 19% within the first three months postpartum [1].

Maternal depression can be treated non-pharmacologically using psychotherapy and/or pharmacologically by prescribing selective serotonin reuptake inhibitors (SSRIs), the most common antidepressive drug therapy during pregnancy [2,3,4,5]. Neonatal abstinence syndrome (NAS) and adverse drug effects have been reported in up to 30% of newborns following SSRI-treatment in late pregnancy [2,5,6,7] and have revealed an increased neonatal morbidity and a higher rate of admissions to the neonatal intensive care unit (NICU) [8].

Sertraline’s major side-effects include (i) agitation, (ii) jitteriness, (iii) irritability, (iv) altered muscle tone, (v) breathing (mostly tachypnea/a respiratory rate >60 per minute and dyspnea), and (vi) suckling problems [2,4,9]. Even adverse effects on early neurological functioning, reflected in general movement (GM) quality, have been reported [10].

The current evidence suggests that adverse side effects or NAS symptoms will last for 1 to 2 weeks after birth, possibly up to 6 weeks [11], and are normally self and time-limiting [2,11].

We here report a severe case of a brief resolved unexplained event (BRUE) [12] in a newborn infant after maternal sertraline treatment during pregnancy.

## 2. Case Report

We are presenting a term male infant delivered via caesarean section with spinal anaesthesia to a 30+-year old woman. By virtue of fear of a spontaneous delivery, a caesarean section was performed. The pregnancy was complicated by gestational diabetes, which was controlled by giving insulin injections. During early pregnancy, maternal depression was diagnosed and treated using sertraline from 20 weeks of gestation onwards, with the dosage being 10 mg daily. Smoking, alcohol and drug consumption was denied. The local ethics committee of the Medical University approved the study and all subjects gave their informed consent for inclusion before they participated in the study. The EK number is 1390/2018.

The delivery and postnatal statuses were normal, with Apgar scores of 9/10/10 at 1, 5 and 10 min after birth. No resuscitation and respiratory support was required. The birth weight was <3rd percentile at 2250 g, length was 45 cm (<3rd percentile), and the head circumference was 33.5 cm (<10th percentile). Criteria for “small for gestational age” were met, which was interpreted as being due to placental insufficiency, identified in a pathological report. Although some agitation was observed during the in-house hospital stay, the physical examinations carried out on days 1 and 5 after birth were found to be normal. At no point was a diagnosis of NAS established, and mother and infant were discharged on day 8. During hospital stay, the infant was breastfed, plus formula additionally.

After being discharged from hospital, a severe apnoic episode in an upright position was observed. During this episode, the infant turned muscular hypotonic and apnoic with pale-grey skin-colour and fell unconscious, which was observed by his mother. According to the mother’s observation, this episode lasted 3 min. Precise stimulation was started by the mother and continued until the ambulance arrived. The infant did not require any chest compressions. The newborn infant was transported to the pediatric emergency room at the University Hospital with a mask oxygen supply.

The examination on admission to the neonatal intensive care unit revealed a vital newborn infant. Assessment included complete and differential blood counts, glucose, electrolytes, inflammatory markers, creatinine and bilirubin, which were all found to be within normal limits. The sertraline serum blood level of the newborn on the day of admission was 0.2 ng/mL, thus falling short of the reference range of 10–150 ng/mL. Sleep laboratory, cerebral ultrasound examination and electroencephalogram were all found to be normal with no further apnoic episodes being observed throughout the remaining hospital stay. The Finnegan score was assessed at a six-hourly frequency with a maximum score of 14, the newborn presenting with some irritability, increased muscle tone, jitteriness, high pitched cry, short sleep after feeding and sneezing and frantic sucking with vomiting and loose stools. The infant was formula-fed after admission and treated with a multimodal therapy approach including non-nutritive sucking, tight swaddling and kangaroo care, but no pharmacological treatment was started. After 7 days, when the NAS symptoms had fully passed, the infant was discharged with a home apnea-monitoring.

## 3. Discussion

Infants exposed to SSRI can experience NAS symptoms within the first few days after birth. NAS symptoms are categorized into four groups including central nervous system and autonomic symptoms (e.g., irritability, altered behaviour), gastrointestinal symptoms (e.g., poor feeding) and respiratory symptoms (e.g., respiratory distress) [2,13,14,15]. Most commonly here, the Finnegan score is used, but also the adapted Finnegan scoring list can be applied to monitor NAS symptoms [16,17]. In the majority of cases, these NAS symptoms disappear within a few days [2,4].

We here report a severe case of a brief resolved unexplained event (BRUE) [12] on day 8 after birth, in association with sertraline exposition during pregnancy. Important other differential diagnoses, e.g., viral or bacterial infections, cerebral abnormalities and haemorrhage, cardiac abnormalities, hypoglycaemia and severe electrolyte alterations have to be excluded primarily. All these clinical investigations were found to be normal in our reported newborn infant.

A database analysis has evaluated 93 cases of SSRI-induced NAS, of which nine were due to sertraline exposure [18]. One infant experienced hypoventilation, and one infant had an apnoic event, but the authors were unable to determine the specific SSRI. In the current case, a serotonin syndrome [5,13], which could be a differential diagnosis to NAS, showing similar symptoms associated with high SSRI plasma concentrations and a postulated direct SSRI effect of serotonin or its metabolites given the symptoms, was excluded with a blood sertraline level (0.2 ng/mL) far below the relevant range. A shortcoming in our report is that we did not have an SSRI blood or plasma level available immediately after delivery because the infant was then admitted somewhere else and presented with an unremarkable first days of life there.

As Klinger and Merlob have pointed out, withdrawal symptoms can also be identified by showing that the timing of peak symptoms does not correspond with the peak exposure, but rather appears with a delay of several days [13]. This also seems to be the situation in the case under discussion here. The average half-life of Sertraline is outlined as 22 h to 36 h, and for its metabolites even longer, at 62 h to 104 h. This pharmacokinetic of sertraline further supports the theory of developing NAS symptoms with a delay of days. Therefore, newborn infants exposed to SSRIs during pregnancy have to be monitored very carefully for up to 4 days (96 h) to recognize the beginning of NAS symptoms and start treatment if necessary.

In the present case, the event occurred on day 8, so the question of a possible influence from breastfeeding arises.

Currently, there are no concerns regarding breastfeeding for SSRI-treated mothers as only low amounts of sertraline are excreted into breast milk [3,9,19] and have not been associated with clear-cut adverse effects in infants [19]. Lanza et al. have reported no adverse effects in infants exposed to sertraline and desmethylsertraline via breastfeeding [20]. Contrary to that, Müller et al. have reported a preterm infant with serotonergic overstimulation, exhibiting muscular hypotonia and decreased reactivity after sertraline intake via breast milk [21]. Because of the wide therapeutic reference range, the interpretation of sertraline serum levels of breastfed newborn infants still remains a challenge. Stowe et al. have suggested discarding breast milk 8 h to 9 h after maternal sertraline ingestion to significantly reduce the infant’s exposure [22]. Although the infant in the current report was additionally formula-fed ever since his birth, we do not know exactly how often the infant received breast milk or formula, or the proportion of the two. Breastfeeding was stopped on admission, which worsened agitation and the Finnegan score increased to 14, which further supports our hypothesis of NAS in the reported infant; in addition, we cannot rule out that the weaning of breastfeeding resulted in a lack of physical comfort.

## 4. Conclusions

Newborn infants exposed to SSRIs during pregnancy should be evaluated closely concerning NAS and monitored for NAS symptoms for a minimum of 72 h to 96 h, or until symptoms fully recover using standardized protocols including the Finnegan score for the evaluation of NAS symptoms. There is a risk of severe NAS symptoms, which should be discussed with the parents prior to administering the drug and taken into account.

## Abbreviations

SSRI	Selective serotonin reuptake inhibitors
NAS	Neonatal abstinence syndrome
BRUE	Brief Resolved Unexplained Events
